# Icariin: A Potential Molecule for Treatment of Knee Osteoarthritis

**DOI:** 10.3389/fphar.2022.811808

**Published:** 2022-04-05

**Authors:** Juntao Zhang, Fangyang Fan, Aifeng Liu, Chao Zhang, Qi Li, Chenglong Zhang, Feng He, Man Shang

**Affiliations:** ^1^ Academy of Medical Engineering and Traditional Medicine, Tianjin University, Tianjin China; ^2^ Orthopedics Department, National Clinical Research Center for Chinese Medicine Acupuncture and Moxibustion, The First Teaching Hospital of Tianjin University of Traditional Chinese Medicine, Tianjin, China; ^3^ Department of Pharmacology, School of Basic Medical Sciences, Tianjin Medical University, Tianjin, China

**Keywords:** icariin, knee osteoarthritis, network pharmacology, molecular docking, molecular mechanism

## Abstract

**Background:** Knee osteoarthritis (KOA) is a degenerative disease that develops over time. Icariin (ICA) has a positive effect on KOA, although the mechanism is unknown. To investigate drug-disease connections and processes, network pharmacology is commonly used. The molecular mechanisms of ICA for the treatment of KOA were investigated using network pharmacology, molecular docking and literature research approaches in this study.

**Methods:** We gathered KOA-related genes using the DisGeNET database, the OMIM database, and GEO microarray data. TCMSP database, Pubchem database, TTD database, SwissTargetPrediction database, and Pharmmapper database were used to gather ICA-related data. Following that, a protein-protein interaction (PPI) network was created. Using the Metascape database, we performed GO and KEGG enrichment analyses. After that, we built a targets-pathways network. Furthermore, molecular docking confirms the prediction. Finally, we looked back over the last 5 years of literature on icariin for knee osteoarthritis to see if the findings of this study were accurate.

**Results**: core targets relevant to KOA treatment include TNF, IGF1, MMP9, PTGS2, ESR1, MMP2 and so on. The main biological process involved regulation of inflammatory response, collagen catabolic process, extracellular matrix disassembly and so on. The most likely pathways involved were the IL-17 signaling pathway, TNF signaling pathway, Estrogen signaling pathway.

**Conclusion**: ICA may alleviate KOA by inhibiting inflammation, cartilage breakdown and extracellular matrix degradation. Our study reveals the molecular mechanism of ICA for the treatment of KOA, demonstrating its potential value for further research and as a new drug.

## Introduction

Knee osteoarthritis (KOA) is a chronic progressive disease with pain, swelling, and even deformity of the knee joint as the main clinical manifestation (Martel-Pelletier et al., 2016; Primorac et al., 2020). OA is the 11th leading cause of disability worldwide, which not only affects patients physically and mentally, but also represents a serious social burden (Palazzo et al., 2016). An epidemiological survey showed that the number of KOA patients in the United States doubled from the 1900’s to the 2000’s (Wallace et al., 2017). According to research in 2010 (Cross et al., 2014), the global standard age prevalence of KOA was 3.8%, and the prevalence increased sharply with age. KOA pathology mainly involves articular cartilage and synovium (Michael et al., 2010). In the physiological state, the cartilage matrix is in a dynamic balance of cytokine-controlled production and breakdown. When mechanically altered or stimulated by inflammation, cartilage degrades and progresses to KOA if the compensatory capacity of the joint is exceeded (Michael et al., 2010). The pathogenesis of KOA, however, is not yet clear. To better guide the clinical treatment of KOA, its pathogenesis needs to be studied urgently.

Icariin (ICA) is one of the main components of the Chinese medicine Epimedium. Epimedium was first recorded in the Yellow Emperor’s Inner Canon in 100 B.C. Traditional Chinese medicine believed that Epimedium could strengthen muscles and bones, and it was widely used in clinical practice. Due to the development of modern medicine, its active ingredients have been further studied. Modern research has discovered that ICA, one of its active ingredients, has strong biological activity on various systems, such as the nervous system (Jin et al., 2019), reproductive system (Zhang et al., 2021), skeletal system (Pan et al., 2017; Wang et al., 2018) and so on. ICA was found to improve bone loss caused by estrogen deficiency through the IGF-1 pathway, the ERK pathway and the JNK pathway (Song et al., 2013; Zhou et al., 2021). It also has the effect of preventing cartilage degradation and promoting cartilage repair, as proved by some *in vivo* and *in vitro* experiments (Wei et al., 2016; Zhang et al., 2019). The most widely used drugs in non-surgical treatment are NSAIDs such as cyclooxygenase-2 (COX-2) inhibitors (Bannuru et al., 2019). However, the long-term use of NSAIDs leads to gastrointestinal complications such as indigestion, gastritis and heartburn (Curtis et al., 2019). Since there is no specific treatment for KOA, there is an urgent need to find a safe and reliable treatment method.

**FIGURE 1:**
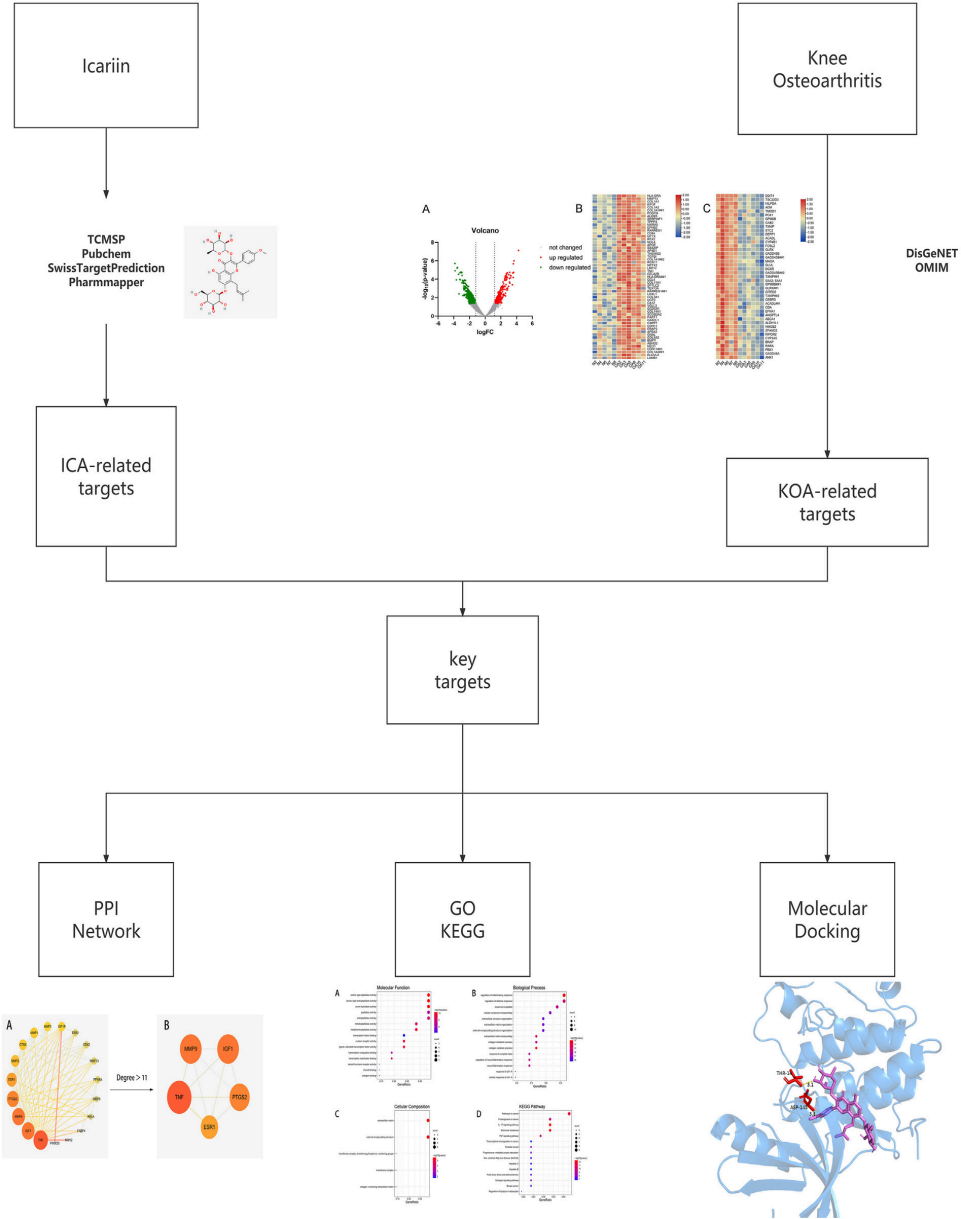
Flow chart of this study based on network pharmacology.

Network pharmacology analysis is one of the most commonly used methods to study the pharmacology of traditional Chinese medicine (TCM). By searching the databases, constructing the networks, enrichment analysis and other steps, we can get the components, potential targets, correlation with diseases and other information of TCM (Yuan et al., 2017). This leads to a more in-depth study of the possible mechanisms of TCM in the treatment of diseases. Molecular docking technology can verify the binding between TCM molecules and predicted targets to validate the accuracy of these targets (Pinzi and Rastelli 2019). Therefore, this study explores the molecular mechanism of ICA for KOA based on network pharmacology, molecular docking, and literature research and the flow chart is shown in Figure 1.

## Materials and Methods

### Collection of Osteoarthritis Related Genes

Firstly, microarray information on differential DNA expression between normal subjects and KOA patients was collected from the GEO database (https://www.ncbi.nlm.nih.gov/geo/) (Edgar et al., 2002), Series GSE169077. The microarray data was normalized with log_2_ (fold change) > 1 or log_2_ (fold change) < -1 as the standard, which was considered to have significant differences in the expression. The KOA related genes were collected by integrating databases through searches with the key words “knee osteoarthritis” and “*Homo sapiens*,” followed by taking the intersection. Two databases include: DisGeNET database (https://www.disgenet.org/web/DisGeNET/menu/home) (Piñero et al., 2017) and OMIM database (https://omim.org/) (Amberger et al., 2015). Next, a visual volcano map and a gene expression heat map were created using Graphpad Prism 8.0.2 (GraphPad Software, San Diego, California United States, www.graphpad.com) and TBtools (https://github.com/CJ-Chen/TBtools) (Chen et al., 2020).

### Potential Target Genes of Icariin in the Treatment of Knee Osteoarthritis

To evaluate the biological information of ICA, it is chemical name, molecular weight, oral bioavailability (OB) and drug likeness (DL) were obtained from the TCMSP database (https://tcmspw.com/tcmsp.php) (Ru et al., 2014). Studies have shown that OB≥40% is considered to have good bioavailability; DL ≥0.18 is considered to be a drug-like compound (Liu et al., 2013). Then the 2D and 3D molecular structures of ICA were obtained through the Pubchem database (https://pubchem.ncbi.nlm.nih.gov/) (Kim et al., 2021).

Next, the potential targets of ICA were predicted using the SwissTargetPrediction database (http://swisstargetprediction.ch/) (Gfeller et al., 2013), Therapeutic Target Database (http://db.idrblab.net/ttd/) (Wang Y. et al., 2020) and Pharmmapper database (http://www.lilab-ecust.cn/pharmmapper/) (Wang et al., 2017), followed by taking intersection set. After that, all targets were converted into gene symbols standardized through the Uniprot database (https://www.uniprot.org/) (Morgat et al., 2020).

### Construction of Protein Interaction Network

The potential targets of ICA were matched with KOA-related genes to obtain the core targets. The venn diagram was drawn through venny 2.1.0 (https://bioinfogp.cnb.csic.es/tools/venny/) (Oliveros 2007-2015). Next, the targets were analyzed by the String database (https://string-db.org/) (Szklarczyk et al., 2019). After that, the PPI network was edited with Cytoscape 3.7.2 software (http://www.cytoscape.org) and the degree and edge betweenness of the nodes were calculated by the NetworkAnalyzer tool.

### Gene Ontology and Kyoto Encyclopedia of Genes and Genomes Enrichment Analysis

Based on the above data, GO and KEGG enrichment analysis was performed in the Metascape database (http://metascape.org/gp/index.html) (Zhou et al., 2019). The Gene Ontology (GO) enrichment analysis includes cellular composition, biological process, and molecular function, whereas the Kyoto Encyclopedia of Genes and Genomes (KEGG) analysis can profile metabolic pathways of genes in the cell as well as their systemic function. Unlike the last update of the David database in 2016, the Metascape database was updated on 1 February 2021. After this, a visual bubble diagram was drawn using the Bioinformatic online tool (http://bioinformatics.com.cn/).

### Construction of Targets-Pathways Network

To investigate the interactions between key genes and pathways of ICA and KOA, the ICA-KOA-targets-pathways network was constructed using the results of metascape database analysis. The degree and edge betweenness were analyzed by topological analysis tools in the cytoscape 3.7.2 software.

### Molecular Docking

Proteins corresponding to the top ten key genes were selected to dock with the ICA molecule to verify their affinity. Crystal structures of proteins were downloaded from the PDB database (https://www.rcsb.org/) (Burley et al., 2021) with the search criteria: *Homo sapiens*, refine resolution<3.0 Å and release date 2015–2021. then we imported the protein structures into PyMOL 2.2.0 software (https://pymol.org) (Schrodinger 2015) for modification including removal of water molecules, separation of ligands and addition of hydrogen. Using AutoDockTools 1.5.6 software (http://autodock.scripps.edu/) (Goodsell and Olson 1990) to add charge to protein molecule and set up a docking grid box at the center of the molecule (Seeliger and de Groot 2010). Molecular docking was performed using AutoDock Vina 1.1.2 software (http://vina.scripps.edu/) (Trott and Olson 2010). We chose it as the software for molecular docking because it is efficient, accurate, and has a new way of evaluation. Finally, after analyzing the binding energy of the molecule, choosing the conformation with the lowest binding energy, and observing the formation of hydrogen bonds, we drew a binding diagram with PyMOL 2.2.0.

### Search and Analysis of Literature

To validate the predictions, we also searched for relevant studies over the last 5 years, including *in vivo* studies, *in vitro* studies and reviews. The search terms we used were “icariin”, “osteoarthritis”, “knee osteoarthritis”, “cartilage”, “chondrocytes”, and “extracellular matrix”. To ensure the relevance of the literature to this study, the results were collated by two independent researchers.

## Result

### Acquisition of Knee Osteoarthritis Related Genes

**FIGURE 2:**
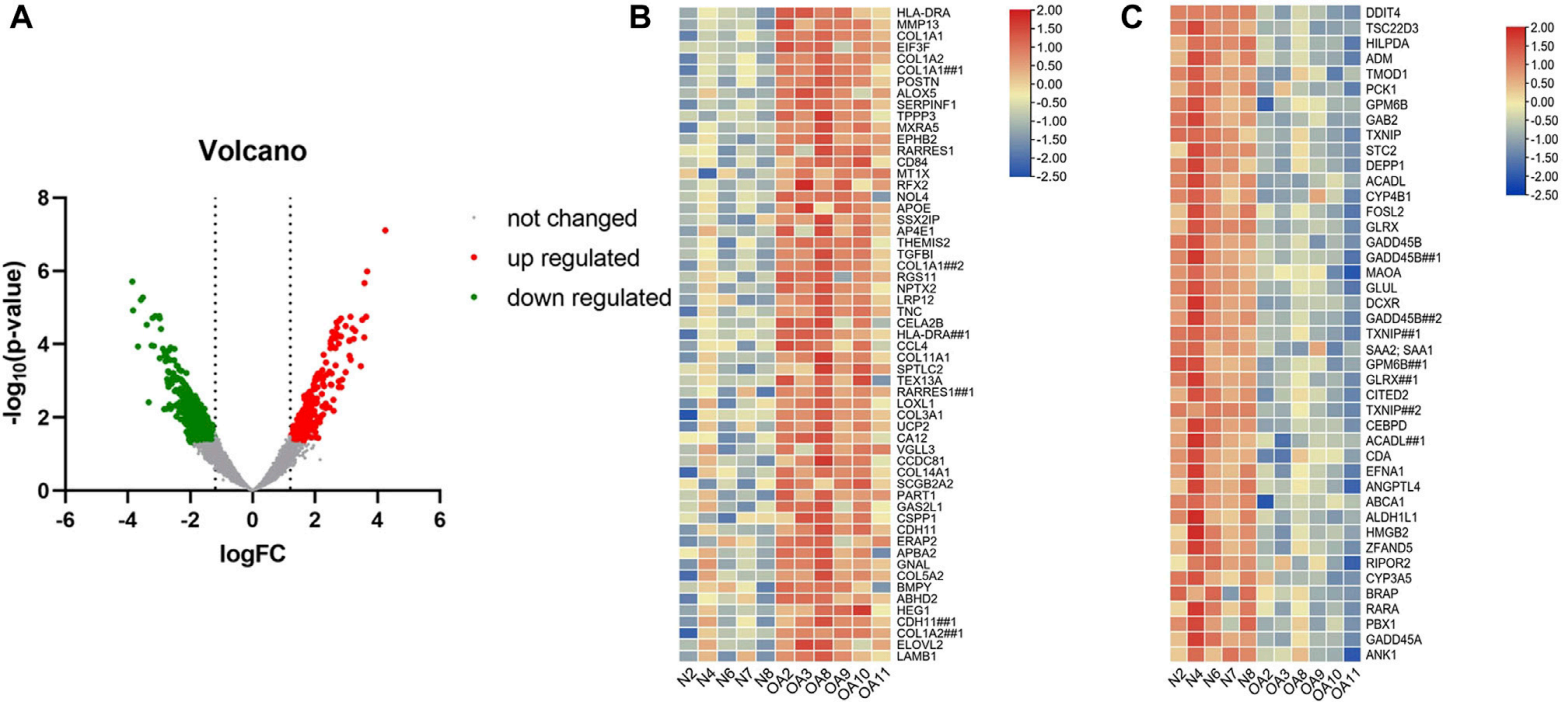
The volcano and heat maps of differential DNA expression in OA patients. **(A)** Red represents up-regulated genes, green represents down-regulated genes, and gray represents unchanged genes. **(B)** The most significantly upregulated genes in cartilage of OA patients versus normal patients. **(C)** The most significantly downregulated genes in cartilage of OA patients versus normal patients.

A total of 1105 differentially expressed genes were identified by analysis of gene microarray Series GSE169077, which was used to draw a volcano may and heat map (Figure 2). The results showed a large difference in gene expression between OA patients and normal subjects. Then 720 out of 9329 genes from the Genecards database (relevance score >4.26), 368 genes from the Disgenet database, 56 genes from the OMIM database, and finally 398 genes were obtained.

### Analysis of Icariin and Collection of its Potential Targets

**FIGURE 3:**
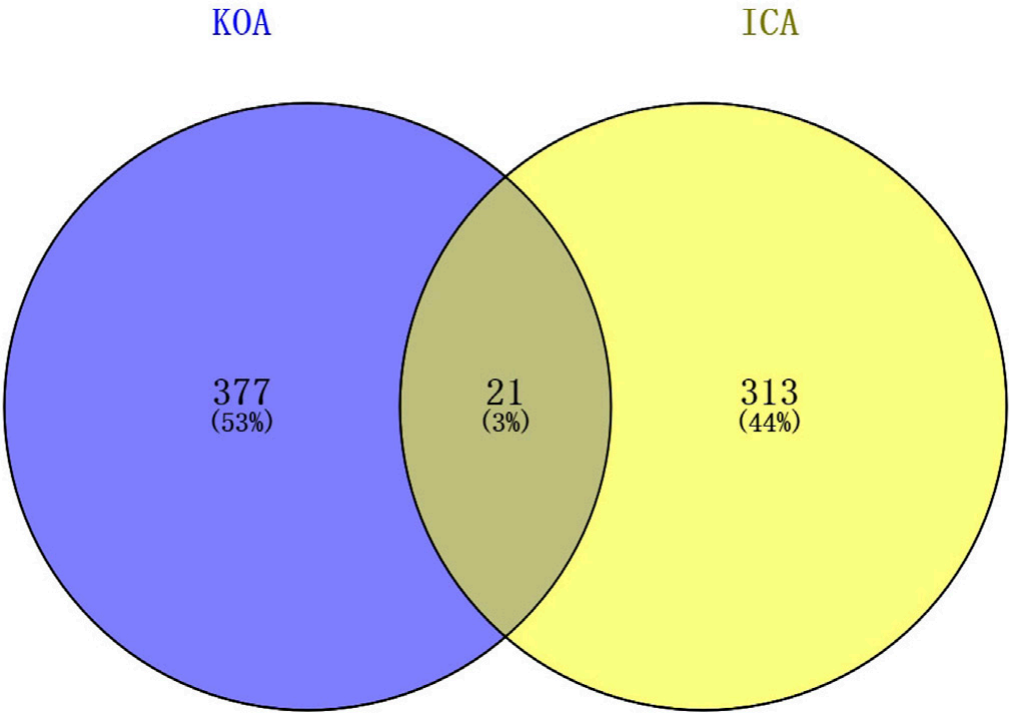
Venn diagram of the targets in KOA and ICA.

Molecular formula, molecular weight, 2D structure, 3D structures, and ADME information of ICA were collected from the TCMSP database and the Pubchem database (Table 1). The OB (41.58%) and DL (0.61) of ICA proved its high bioavailability and drug-like property, which can be used as a drug molecule for the next step of research. A total of 334 potential targets were obtained by combining and removing duplicate ones from the SwissTargetPrediction database, the Pharmmapper database, and the Therapeutic Target Database. Next, the ICA targets were intersected with the KOA genes to obtain the key genes and plotted a Venn diagram (Figure 3).

**TABLE 1:** Basic information of ICA.

Molecular Name	Molecular Formula	Molecular Weight	2D Structure	3D Structure
Icariin	C_33_H_40_O_15_	676.66 g/mol	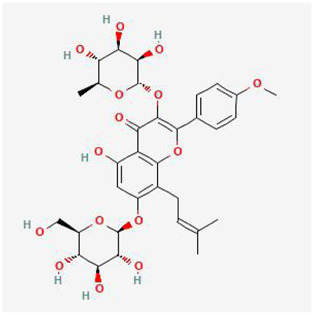	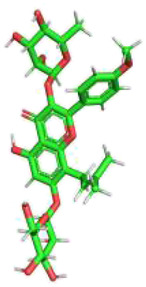

### Protein-Protein Interaction Network Analysis

**FIGURE 4:**
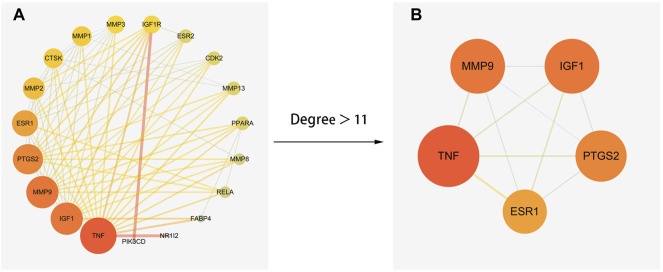
The PPI network of ICA and KOA. **(A)** the network with total 18 nodes and 78 edges. **(B)** the core network with 5 nodes and 7 edges.

The obtained 21 common genes were uploaded to the String database to produce the PPI network. After that, the results were imported into cytoscape 3.7.2 software and the topological parameters of the network was analyzed. The result showed that the network had a total of 18 nodes and 78 edges (Figure 4A). According to the analysis results, TNF was the most important target in the network (Degree 17, BC 0.24, CC 0.95). To find the key genes of ICA in treatment of KOA, a core network with 5 nodes and 10 edges was filtered by Degree>11 (Figure 4B). The other 4 genes were MMP9, IGF1, PTGS2 and ESR1. Finally, the 5 core genes screened by the NetworkAnalyzer tool and their Degree values and Betweenness Centrality values were shown in Table 2.

**TABLE 2:** Topological information of key targets.

Target	Degree	Betweenness Centrality	Closeness Centrality
TNF	17	0.235	0.947
IGF1	15	0.101	0.857
MMP9	15	0.080	0.857
PTGS2	14	0.070	0.818
ESR1	12	0.035	0.750

### Gene Ontology and Kyoto Encyclopedia of Genes and Genomes Enrichment Analysis

**FIGURE 5:**
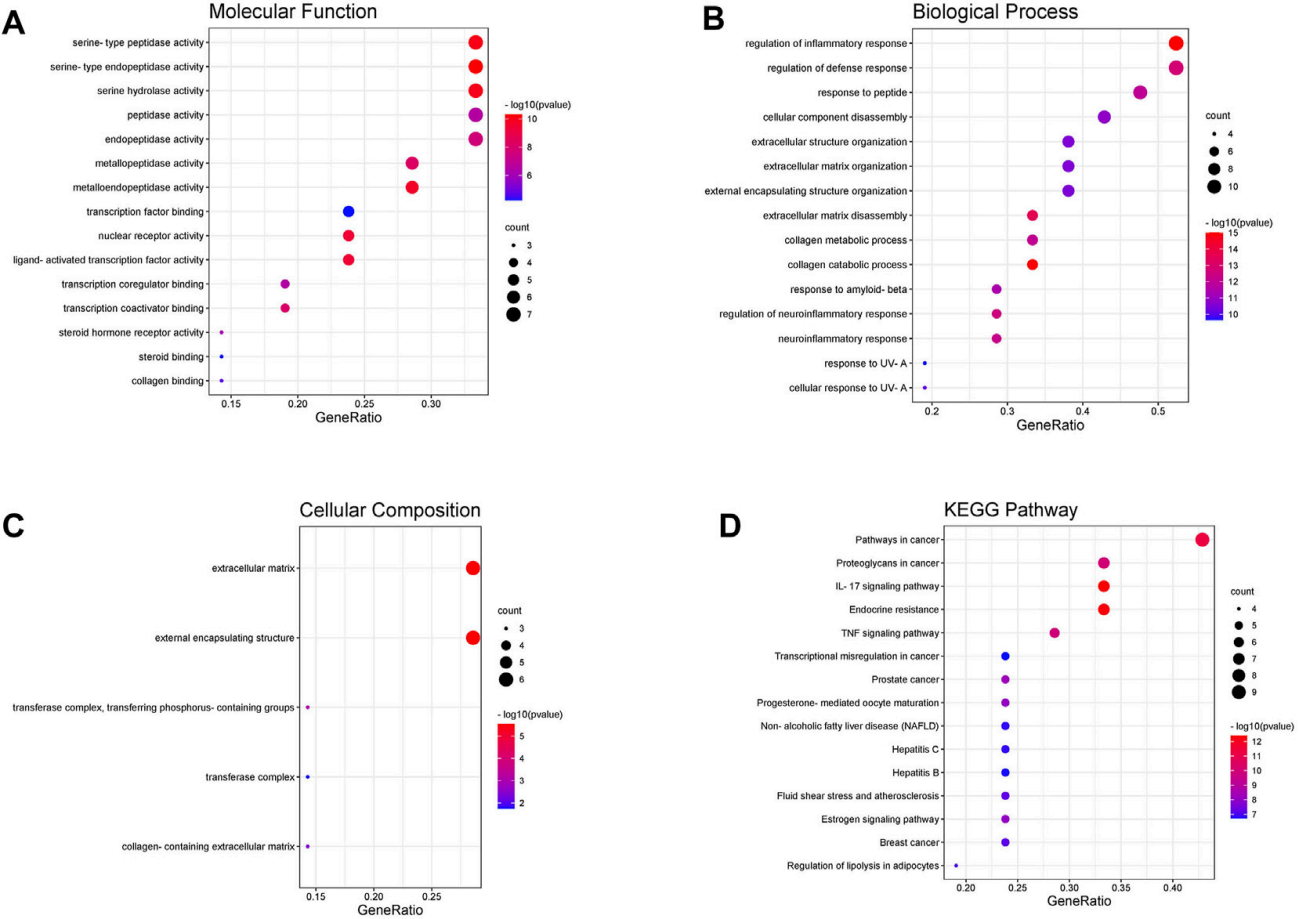
The GO and KEGG enrichment analysis of key targets. Including **(A)** cellular components, **(B)** molecular functions, **(C)** biological processes and **(D)** KEGG pathway analysis.

**FIGURE 6:**
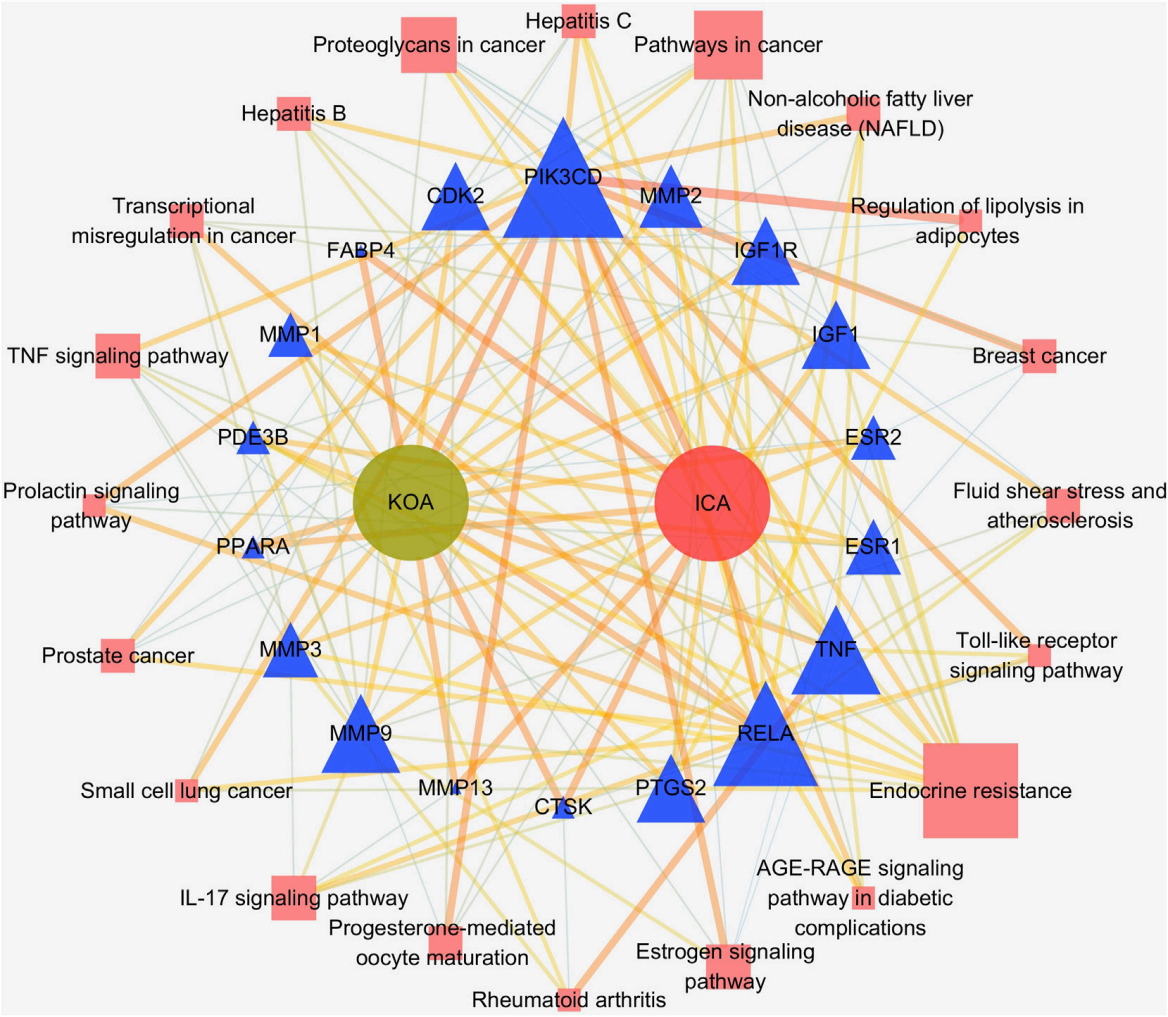
The targets-pathways network of ICA and KOA. Blue triangles represent ICA and KOA core genes, pink squares represent pathways. The size of the shapes represents the value of Degree.

351 GO items were collected through the analysis of the Metascape database (p 0.01, count 3), while the KEGG entries were 112 (p 0.01, count 3). The top 15 most significant entries were filtered in ascending *p*-value order and displayed in a visual bubble diagram, and all results were shown if less than 15 (Figure 5). The results show that the most significant molecular function included serine-type endopeptidase activity, serine-type peptidase activity, metallopeptidase activity, nuclear receptor activity and so on. While the biological process was primarily involved in processes such as collagen catabolism, extracellular matrix disassembly, collagen metabolic process, and other extracellular matrix-related processes. Another aspect is the processes associated with inflammation, such as regulation of inflammatory responses, regulation of neuroinflammatory responses and so on. KEGG results showed that genes shared by ICA and KOA were mainly enriched in the IL-17 signaling pathway, TNF signaling pathway and Estrogen signaling pathway, excluding other disease pathways (Figure 5D; Table 3). Then a targets-pathways network was constructed based on the results (Figure 6).

**TABLE 3:** Top 3 KEGG pathways and related targets, excluding other disease.

Term	Pathway	Key Targets in the Pathway	*p* Value
ko04657	IL-17 signaling pathway	MMP1,MMP3,MMP9,MMP13,PTGS2,RELA,TNF,PIK3CD,MMP2,PPARA,CDK2,ESR1,ESR2,CTSK,PDE3B,RORA	3.8E-13
hsa04668	TNF signaling pathway	MMP3,MMP9,PIK3CD,PTGS2,RELA,TNF	1.4E-10
ko04915	Estrogen signaling pathway	ESR1,ESR2,MMP2,MMP9,PIK3CD,MMP1	9.0E-09

### Molecular Docking Analysis

**FIGURE 7:**
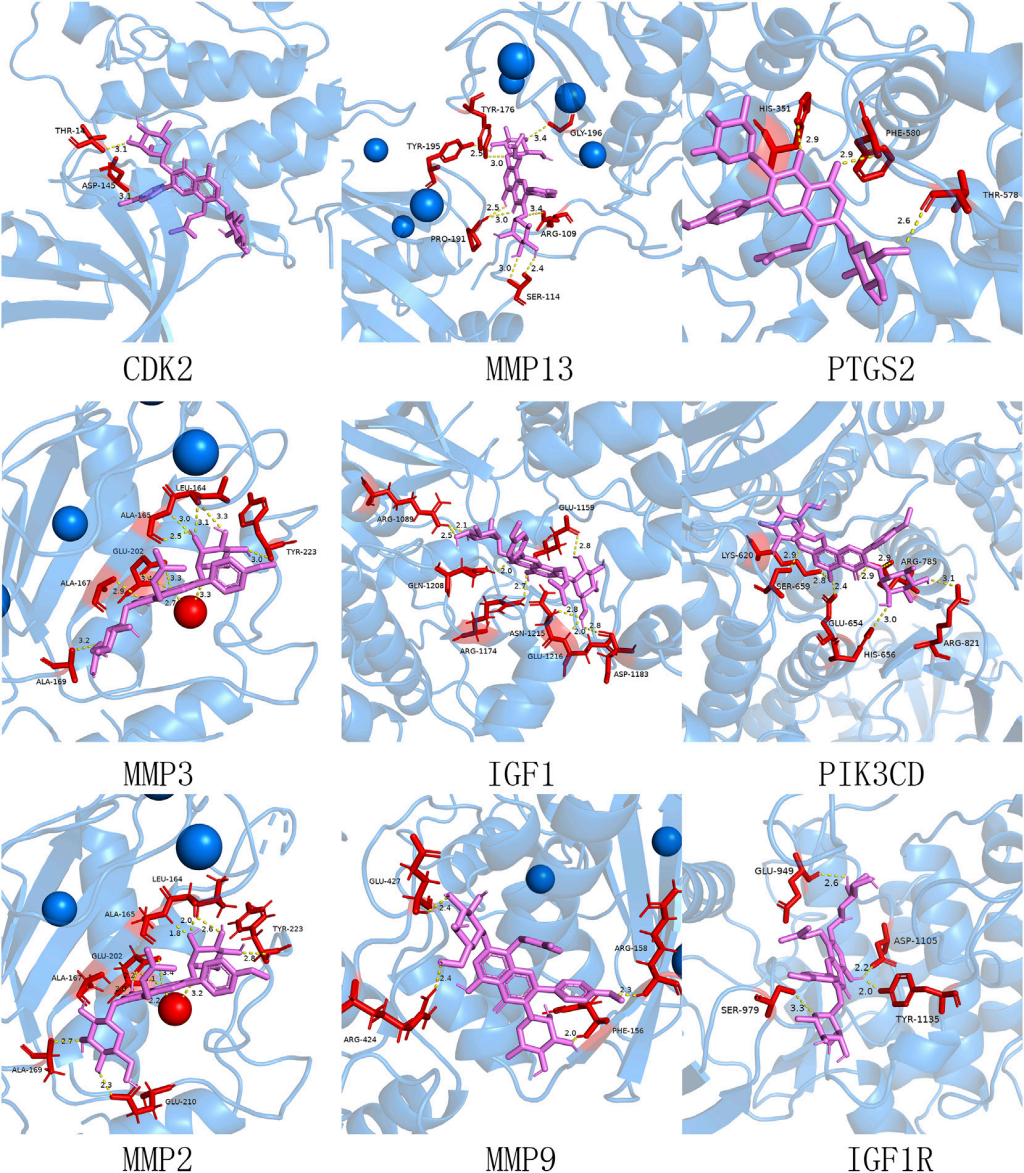
Molecular docking simulation diagram. The ICA is violet stick models and the protein molecules are blue cartoon models. The protein molecules at the docking site are represented as red stick models. The connected hydrogen bonds are indicated by yellow dotted lines. And the blue and red bubbles indicate metal ions.

Based on the data above, the 21 core target genes were individually imported into the Autodock Vina 1.1.2 software to dock with the ICA. Affinity was the score used by the software to calculate the binding ability. It is generally believed that affinity < −7 kcal/mol indicated a stronger binding activity (Ferreira et al., 2015; Gromiha et al., 2017; Cui et al., 2020). After calculation, it was found that 17 out of 21 genes met the criteria of affinity < −7 kcal/mol (Table 4). The docking diagrams of ICA and genes were drawn using Pymol software (Figure 7). In addition, we found the hydrogen bonds formed between molecules and represented by yellow dotted lines in the diagrams.

**TABLE 4:** Affinity of gene binding to ICA.

The Targets	Affinity (kcal/mol)	The Targets	Affinity (kcal/mol)
CDK2	−10.1	ESR1	−8.2
MMP13	−9.5	PDE3B	−8.2
PTGS2	−9.5	ESR2	−8.0
MMP3	−9.1	CTSK	−8.0
IGF1	−9.1	TNF	−7.7
PIK3CD	−8.8	RELA	−7.5
MMP2	−8.8	MMP1	−7.4
MMP9	−8.4	PPARA	−7.2
IGF1R	−8.3		

### Results of the Literature Collection

**FIGURE 8:**
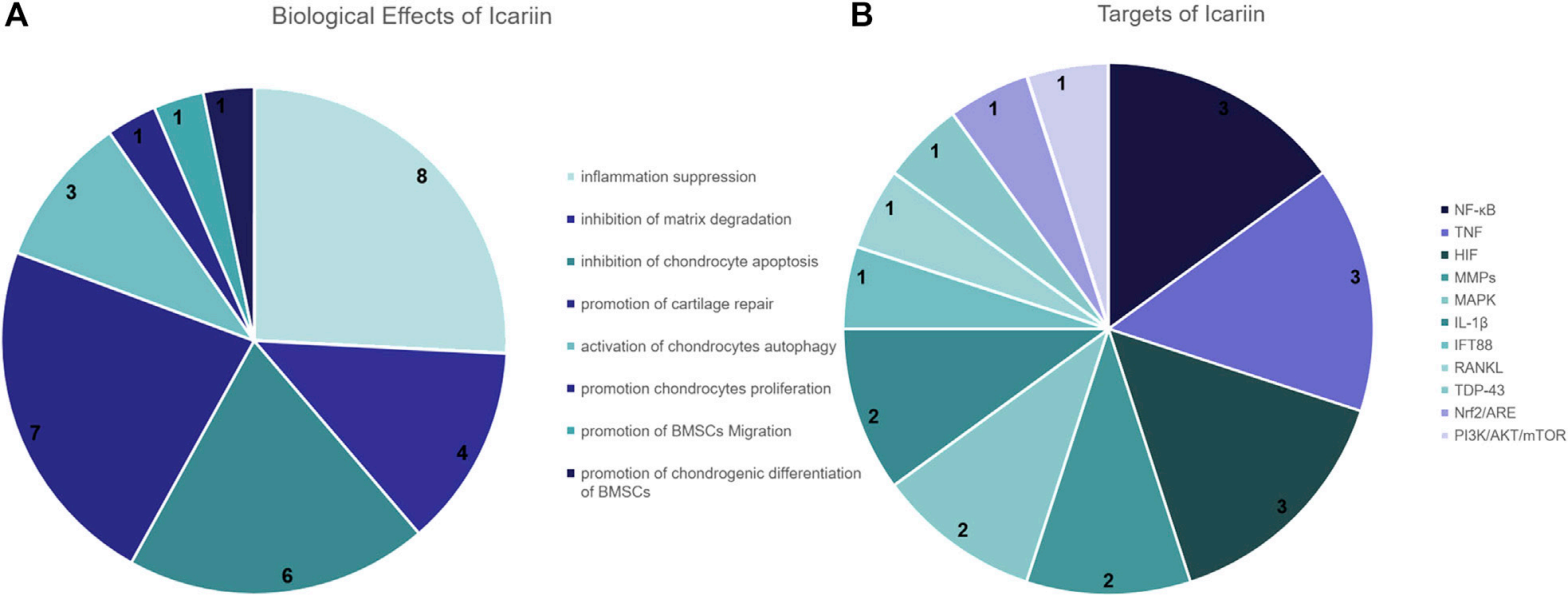
Pie chart of icariin-related literature studies. **(A)** Number of times icariin has been mentioned in the literature for the treatment of KOA effects. **(B)** Number of icariin targets mentioned in the literature.

Through our study of the literature, we screened twenty-eight articles from the last 5 years relevant to this study. Of these, 8 papers mentioned that icariin inhibited inflammation, 6 papers indicated that icariin reduced chondrocyte apoptosis and 4 papers indicated that icariin inhibited extracellular matrix degradation. In addition, 9 papers mentioned that icariin promotes chondrocyte proliferation, differentiation, cellular autophagy or cartilage repair (Figure 8A). In the literature on the underlying mechanisms, 3 papers suggested that icariin can treat OA via the NF-κB signaling pathway, 3 papers mentioned that icariin inhibited the expression of TNF-α, 3 papers mentioned that icariin downregulated the expression of MMPs, while 2 papers mentioned that icariin reduced the inflammatory response caused by IL-β (Figure 8B).

## Discussion

Despite the significant developments in the diagnosis and treatment of KOA, single drug development for a single target is still slow (Wang et al., 2015). This is because KOA is a complex pathological process involving multiple substances and regulated by multiple pathways (Abramoff and Caldera 2020). Coincidentally, traditional Chinese medicine has a multi-target and multi-pathway biological effect. As a traditional Chinese medicine, epimedium is widely used in clinical practice. It has also been widely used in the treatment of knee osteoarthritis from ancient times to the present day with good healing properties. As research progressed, ICA, one of the main components of Epimedium, was found to have strong biological activity. Due to its low price and easy availability, it has a high potential value in the treatment of KOA. In the present study, based on network pharmacology, we found that icariin has good bioavailability. Meanwhile, we assembled a number of key targets and pathways, as well as biological functions, and reviewed the literature over the last 5 years to validate them.

### Icariin May be Useful for the Treatment of Knee Osteoarthritis by Inhibiting Inflammation

Inflammation is a major factor in the development and progression of KOA. On the one hand, inflammatory mediators promote cartilage apoptosis and extracellular matrix degradation. On the other hand, the products of chondrocyte apoptosis and matrix breakdown induce the production of inflammatory substances that further aggravate the KOA condition (Martel-Pelletier et al., 2016). These inflammatory mediators include tumour necrosis factor (TNF), interleukin (IL), nitric oxide (NO), prostaglandin E2 (PGE_2_) and so on (Fei et al., 2019; Li et al., 2020).

**FIGURE 9:**
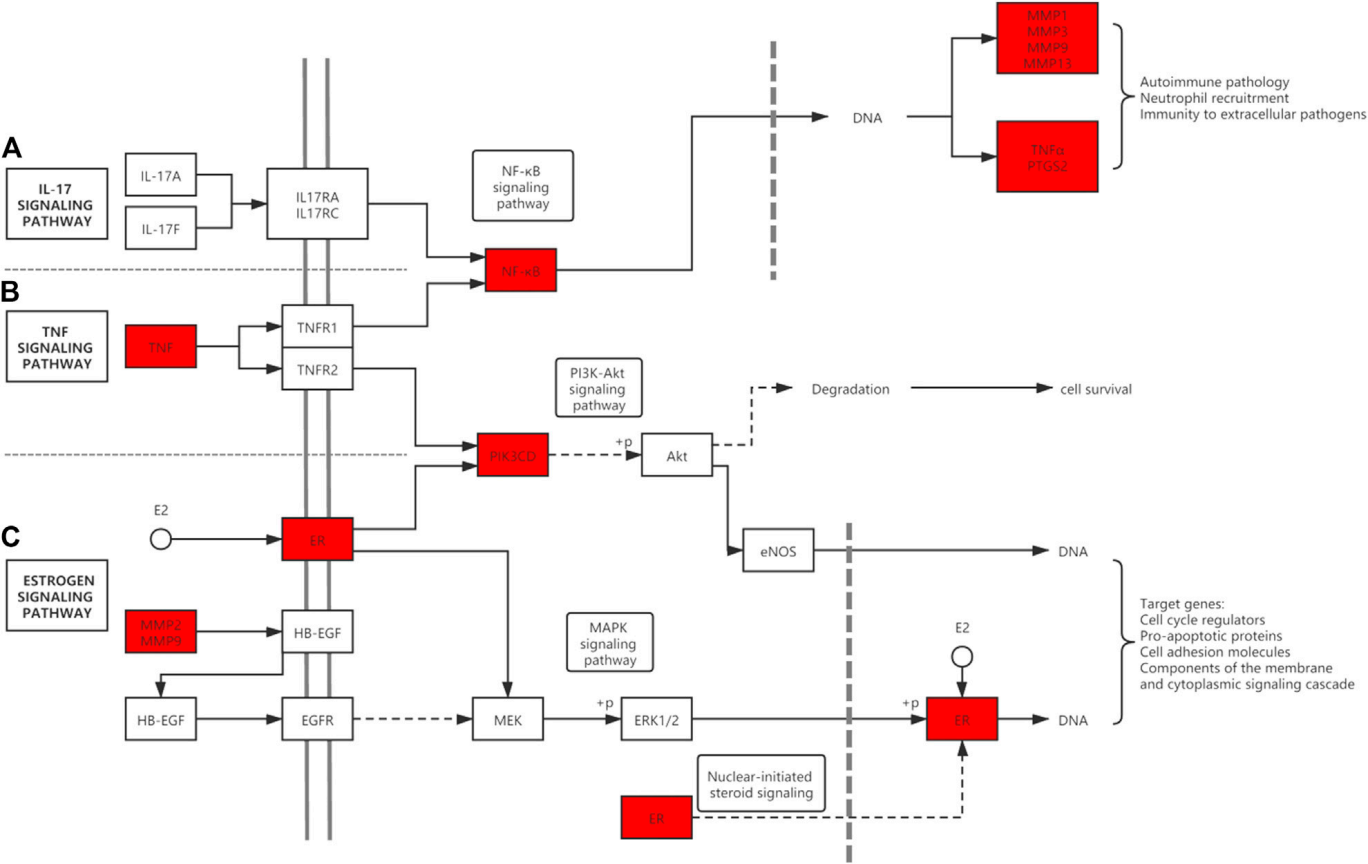
Diagram of ICA potential target genes and pathways. ICA targets are shown in red. **(A)** The IL-17 signaling pathway. **(B)** The TNF signaling pathway. **(C)** The Estrogen signaling pathway. There are also cross talks among pathways.

Our study found that the key targets of icariin for the treatment of KOA include TNF, PTGS2 as well as RELA (NF-κB). TNF is a powerful pro-inflammatory factor that binds to two types of receptors, including TNFR1 and TNFR2, which are expressed on the surface of both chondrocytes and synovial cells (Ansari et al., 2020) (Figure 9). TNF binding to the receptor activates the downstream NF-κB signaling pathway, thereby up-regulating the synthesis of inflammatory mediators (PTGS2, NO, PGE_2_, IL-6, etc.) and matrix degrading enzymes (MMPs, ADAMTS). In addition, it can inhibit the synthesis of type II collagen (Wojdasiewicz et al., 2014). A study reported that icariin can inhibit NF-κB activation and nuclear translocation triggered by TNF-α, as well as reduce chondrocyte apoptosis and inflammation (Mi et al., 2018). In addition, according to the study of Pengzhen Wang et al. (2020), icariin inhibited the activation of NF-κB and HIF-2α in the chondrocytes of OA mice and attenuated the inflammatory response driven by TNF-α. In addition, the expression of MMPs was inhibited. These findings confirm that icariin reduces KOA inflammation by down-regulating the TNF signalling pathway through inhibition of TNF-α, NF-κB. In addition to the above proven targets, we also identified PTGS2 as a potential target for icariin. This could be an area for further research.

Icariin may contribute to the treatment of KOA by promoting cartilage repair and attenuating cartilage extracellular matrix degradation.

In KOA, chondrocyte apoptosis and extracellular matrix degradation are severe pathological changes (Choi et al., 2019). The extracellular matrix is mostly made up of type 2 collagenase and aggrecan, which can be degraded by matrix-degrading enzymes like MMPs and ADAMTS (Guilak et al., 2018). And this is associated with the accumulation of inflammatory substances in KOA joints including TNF-α, IL-6, IL-1β (Krishnan and Grodzinsky 2018). Degradation of the extracellular matrix promotes chondrocyte apoptosis at the same time (Komori 2016).

Our research has found substantial evidence that icariin can promote cartilage repair in KOA over the last 5 years (Wang et al., 2016; Liu et al., 2019; Zhang et al., 2019). Other studies have shown that icariin regulates the TDP-43 signaling pathway and NF-κB signaling pathway to reduce chondrocyte apoptosis (Mi et al., 2018; Huang et al., 2019). In addition, Tang Y, et al. showed that icariin mediated the PI3K/AKT/mTOR pathway to activate chondrocyte autophagy (Tang et al., 2021).

In addition to the above findings, we found that insulin-like growth factor 1 (IGF-1) is also one of the key targets of icariin. It is closely related to the proliferation, differentiation and maintenance of the phenotype of cartilage (Oh and Chun 2003). One study found that icariin enhances IGF-1 signalling, promotes osteogenic differentiation and has estrogen-like effects (Zhou et al., 2021). This suggests that icariin promotes chondrocyte proliferation and differentiation via IGF-1, thereby repairing KOA-damaged cartilage.

We also found that icariin also binds to the estrogen receptors and activates estrogen signaling pathway (Figure 9). The estrogen signaling pathway also plays a significant role in the skeletal system. In particular, post-menopausal women have a high incidence of arthritis and cardiovascular disease. Estrogen was found to have good anti-inflammatory effects and reduce cartilage damage (Martín-Millán and Castañeda 2013). This may be one of the reasons why there are more KOA patients among females than males over the age of 50.

Network pharmacology as a new technology has a wide range of applications in exploring the molecular mechanisms of Chinese medicine. In this study, we predicted the molecular mechanism of ICA for the treatment of KOA and obtained 21 relevant targets. Further analysis obtained the core targets as TNF, MMP9, IGF1, PTGS2, ESR1 and so on. After GO and KEGG analysis, we found that the key pathway for ICA treatment of KOA is the IL-17 signaling pathway, TNF signaling pathway and Estrogen signaling pathway with a regulatory effect on the extracellular matrix degradation and inflammation. Molecular docking verified the affinity of key targets. Finally, we reviewed the literature related to icariin for treatment of KOA in the last 5 years, and the results are generally consistent with the prediction.

## Conclusion

Taken together, ICA is able to inhibit KOA inflammation, chondrocyte apoptosis, matrix degradation, and promote cartilage repair through multiple pathways at multiple targets. Through network pharmacology and molecular docking technology, we obtained the interaction relationship between genes, protein molecules and revealed the molecular mechanism of ICA for the treatment of KOA, making it possible as a new drug.
