# Supplementary figures and images for: Icariin: A Potential Molecule for Treatment of Knee Osteoarthritis
Source: Front Pharmacol. 2022 Apr 5;13:811808. doi: 10.3389/fphar.2022.811808 (PMC9037156; doi:10.3389/fphar.2022.811808)

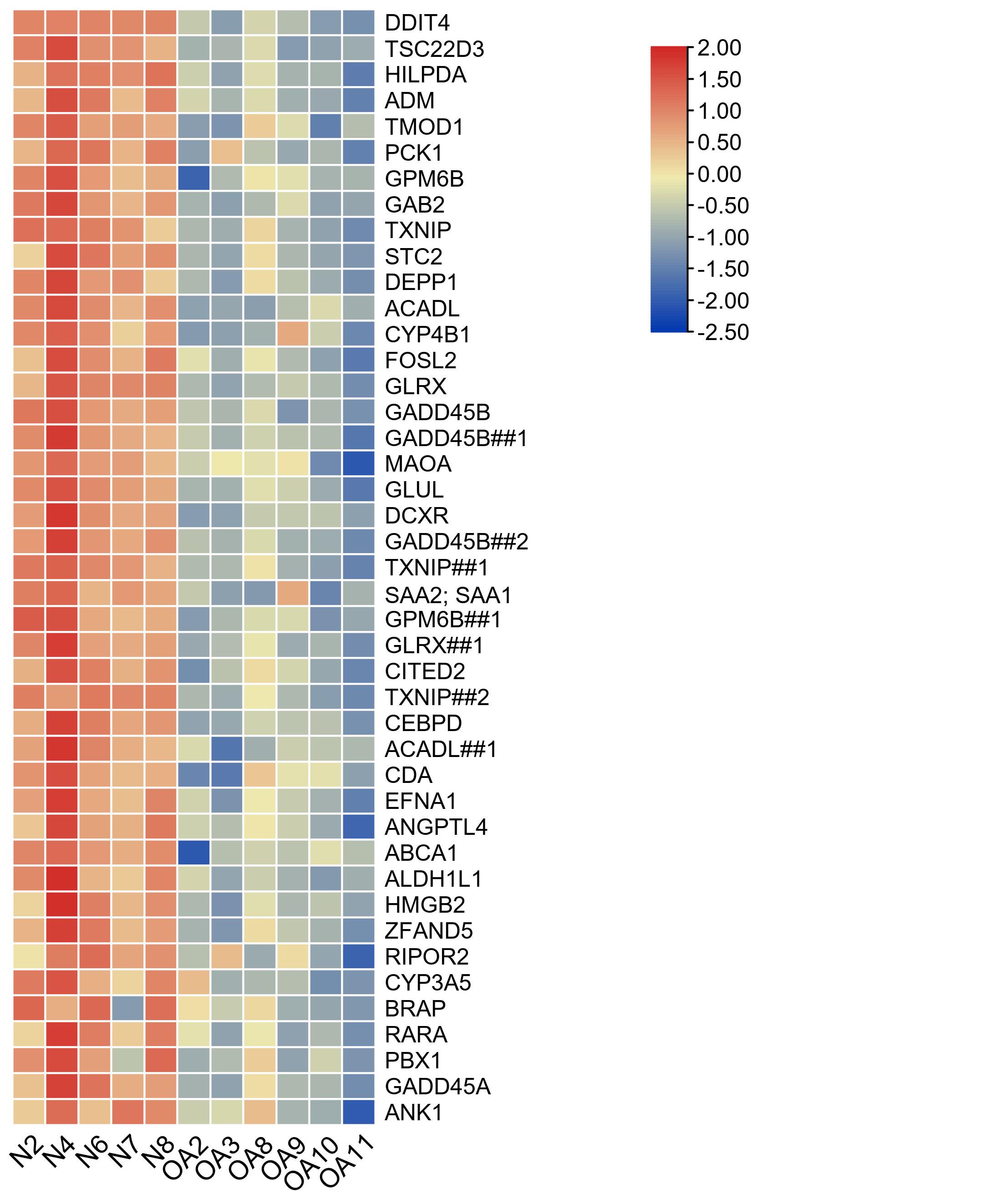

Supplement: Supplementary file 2 [file Image3.JPEG]

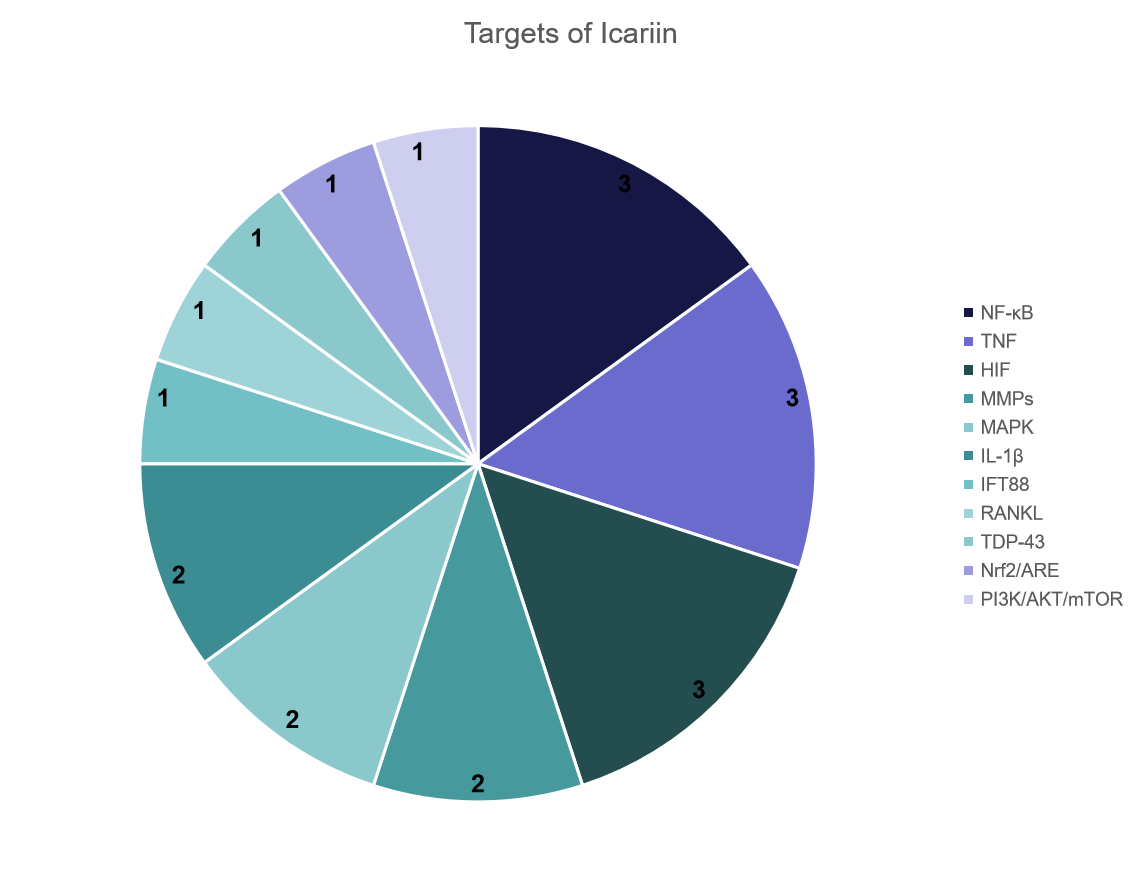

Supplement: Supplementary file 3 [file Image11.PNG]

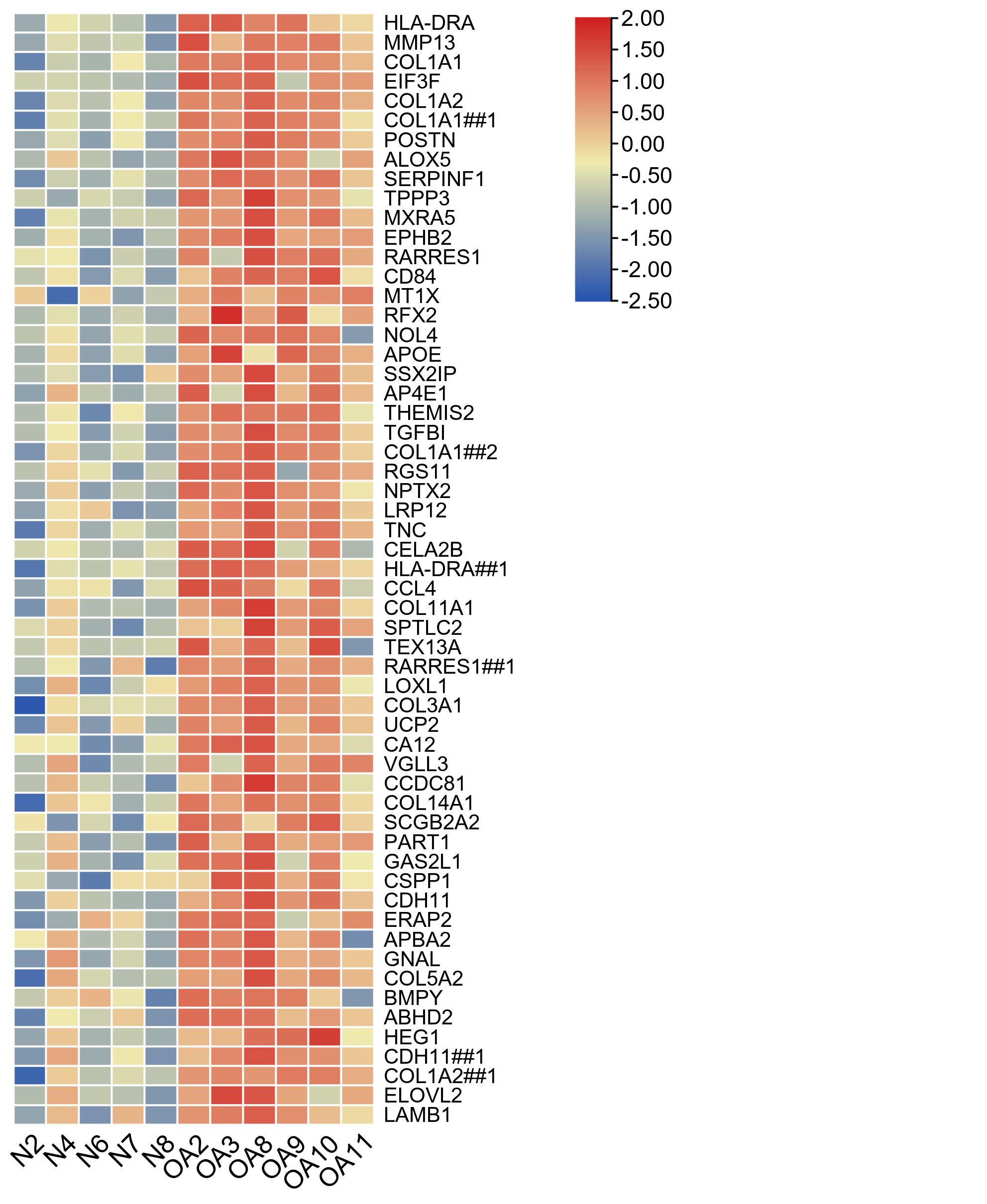

Supplement: Supplementary file 4 [file Image2.JPEG]

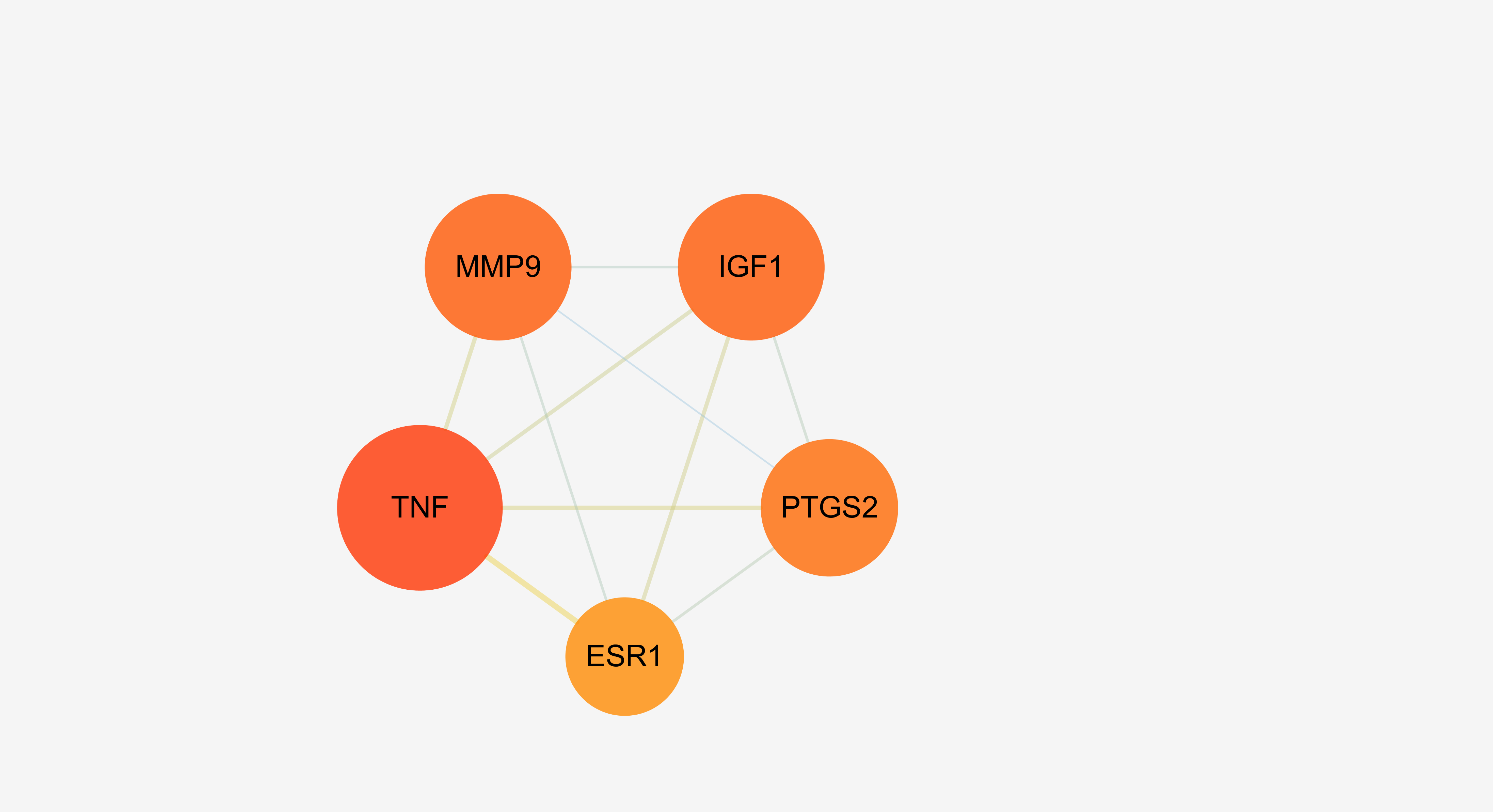

Supplement: Supplementary file 5 [file Image5.PNG]

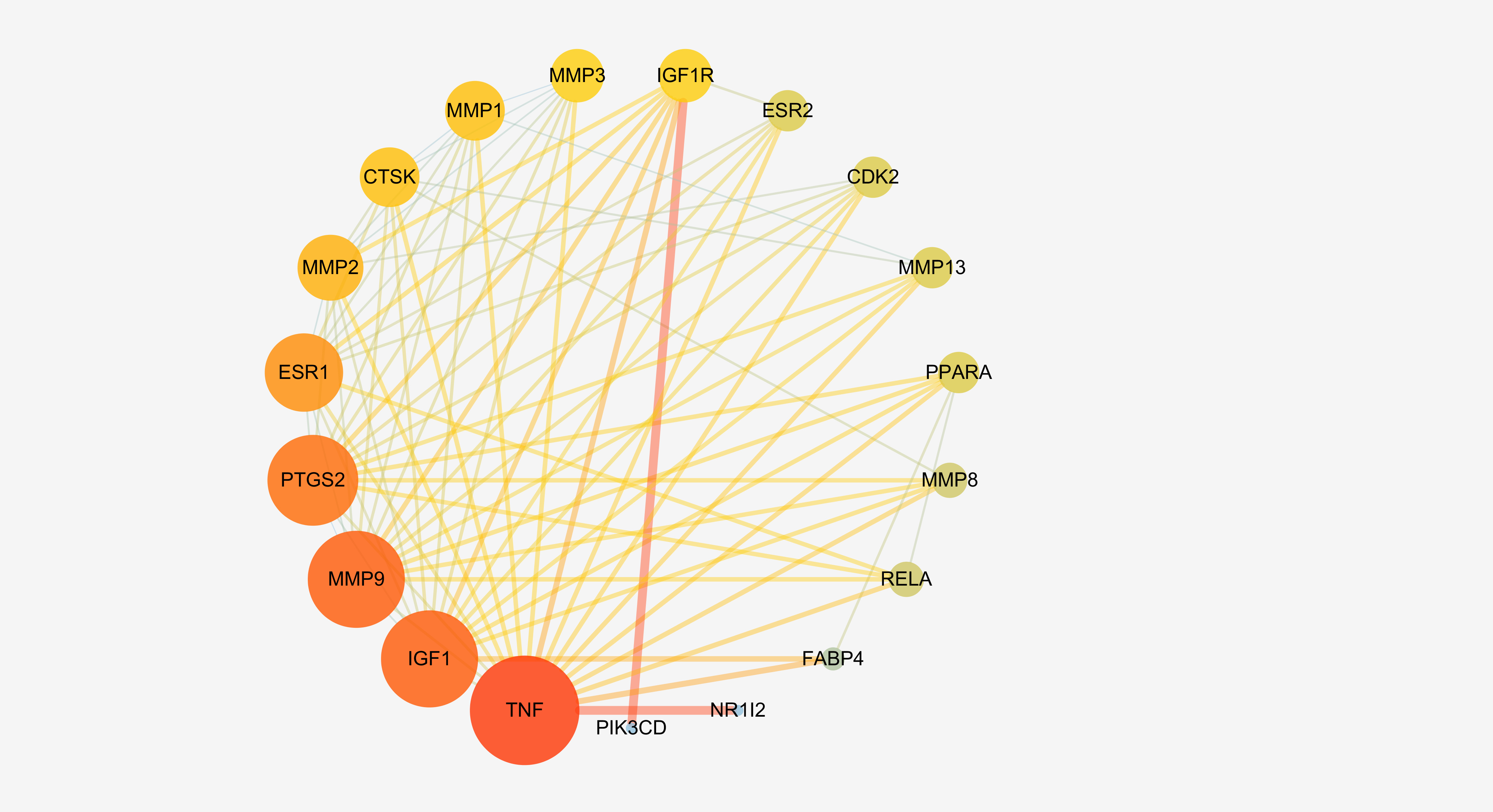

Supplement: Supplementary file 6 [file Image4.PNG]

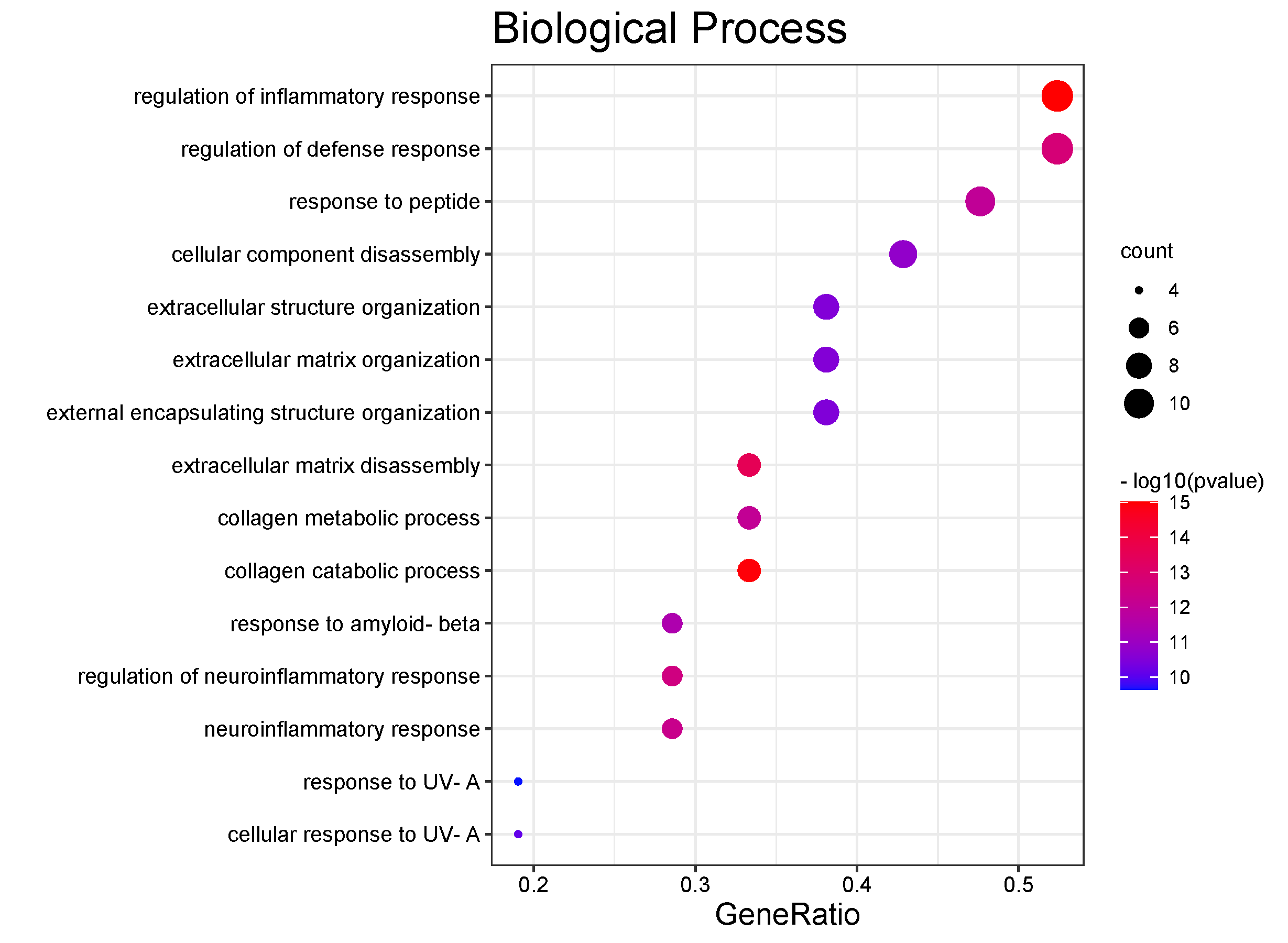

Supplement: Supplementary file 7 [file Image7.PNG]

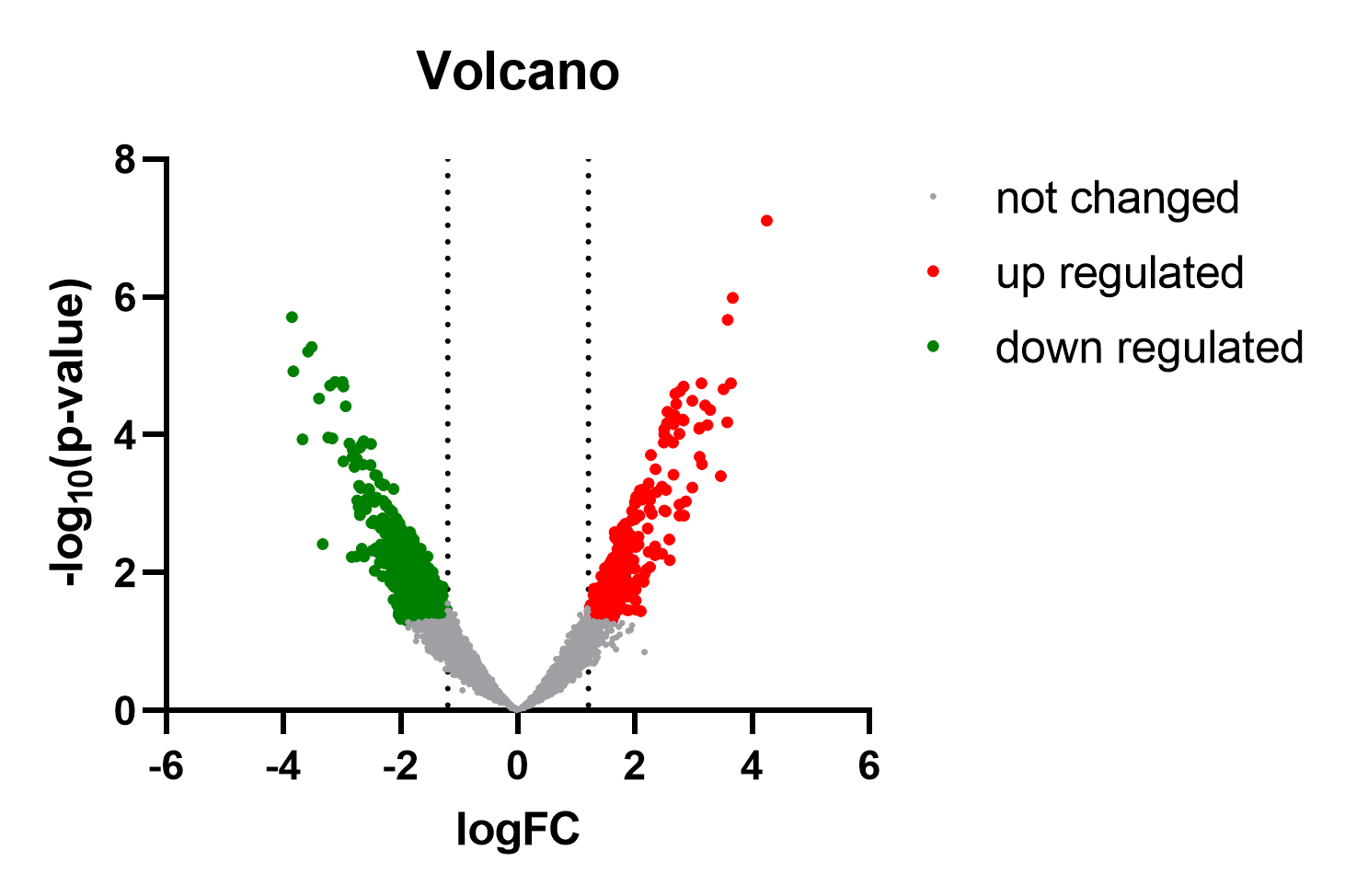

Supplement: Supplementary file 9 [file Image1.PNG]

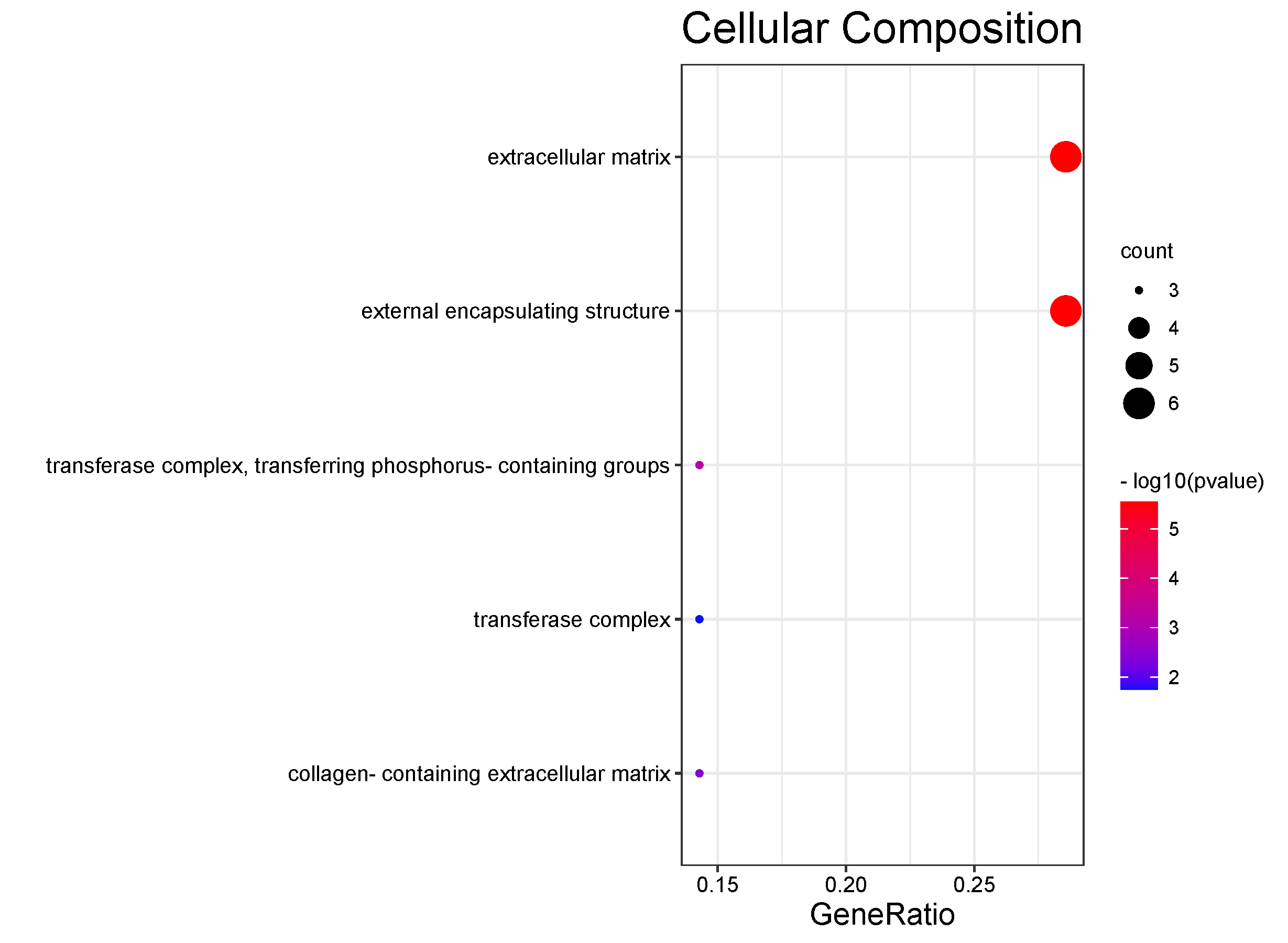

Supplement: Supplementary file 10 [file Image8.PNG]

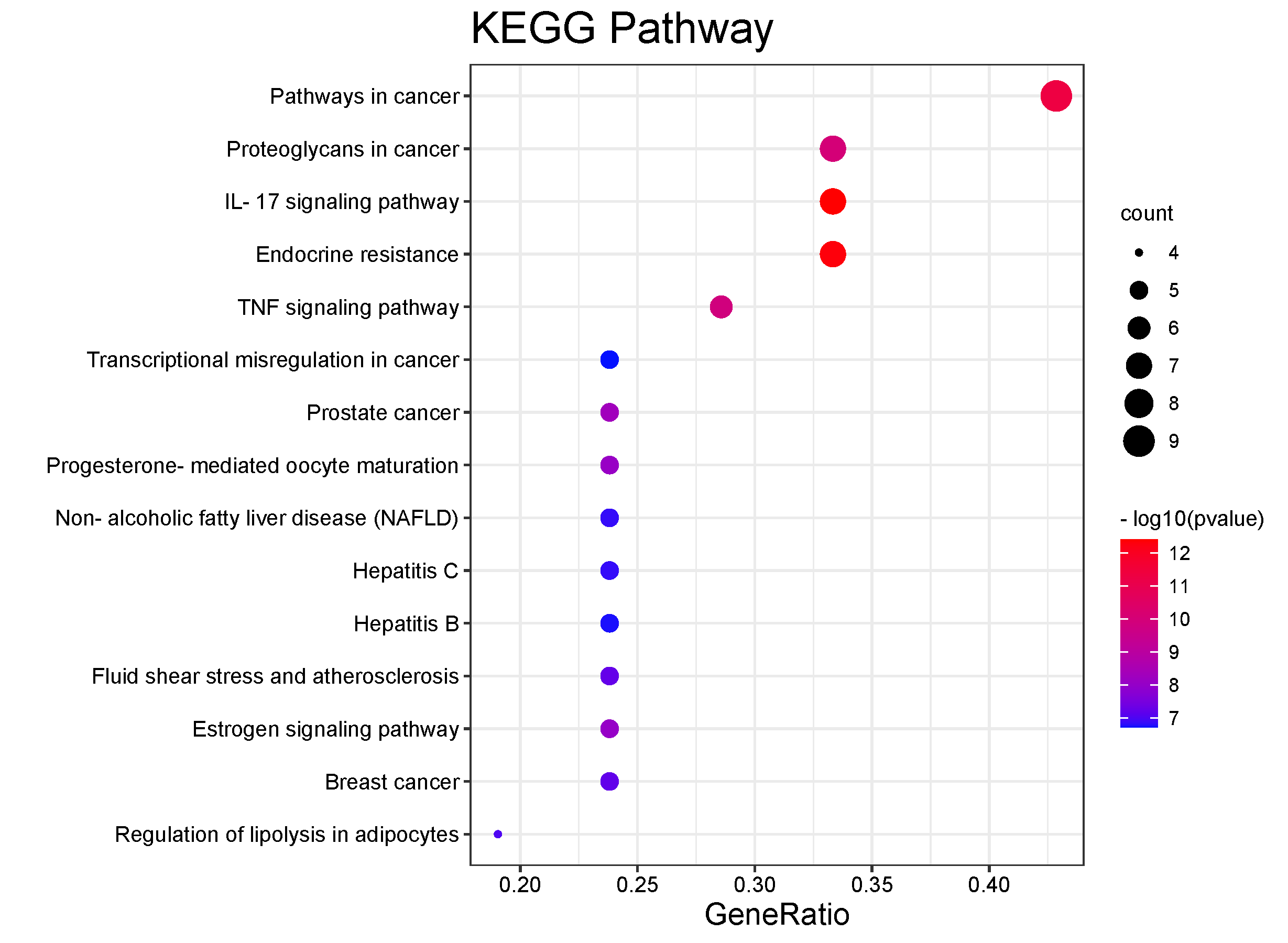

Supplement: Supplementary file 11 [file Image9.PNG]

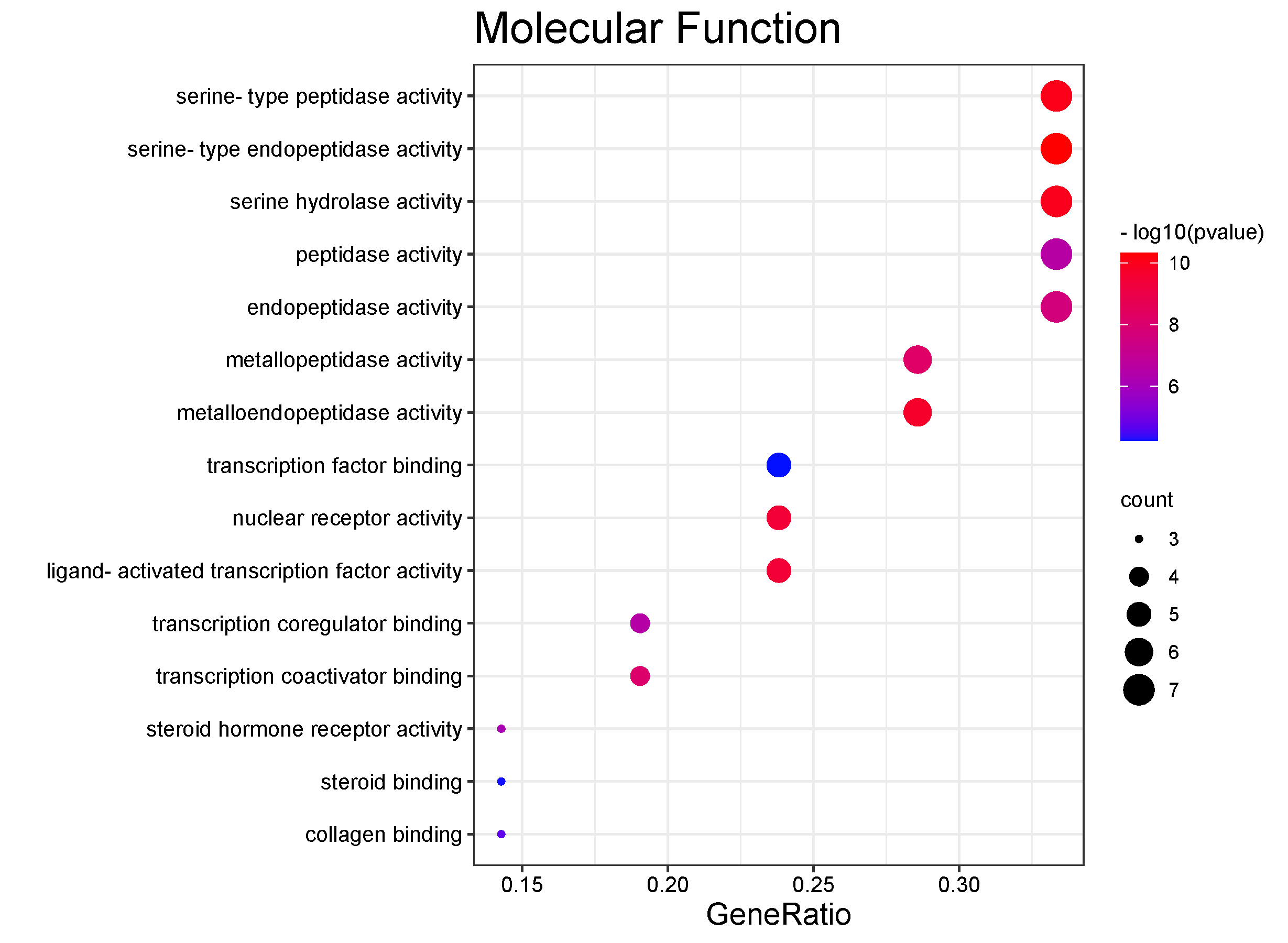

Supplement: Supplementary file 12 [file Image6.PNG]

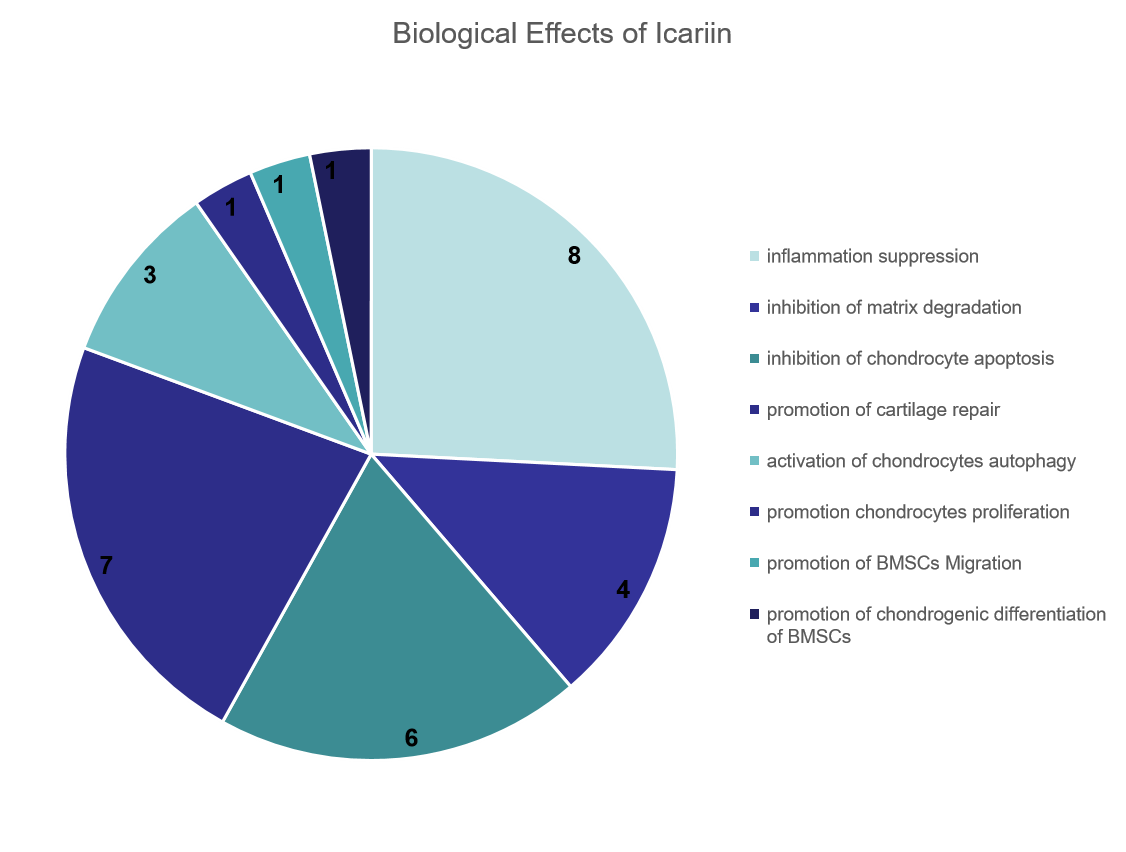

Supplement: Supplementary file 13 [file Image10.PNG]
